# SARS-CoV-2-IgG-Antikörperseroprävalenz bei Personal in der außerklinischen Bekämpfung der COVID-19-Pandemie

**DOI:** 10.1007/s10049-021-00948-z

**Published:** 2021-10-12

**Authors:** Bastian Brune, Johannes Korth, Kai Fessmann, Daniel Stappert, André Nohl, Thomas Lembeck, Fabian Standl, Andreas Stang, Ulf Dittmer, Oliver Witzke, Anke Herrmann, Marcel Dudda

**Affiliations:** 1 Ärztliche Leitung Rettungsdienst, Feuerwehr Essen, Essen, Deutschland; 2 Klinik für Hand‑, Unfall- und Wiederherstellungschirurgie, Universitätsmedizin Essen, Universitätsklinikum Essen, Hufelandstr. 55, 45147 Essen, Deutschland; 3 Klinik für Nephrologie, Universitätsmedizin Essen, Universitätsklinikum Essen, Essen, Deutschland; 4 Feuerwehr Essen, Essen, Deutschland; 5 Ärztliche Leitung Rettungsdienst, Feuerwehr Oberhausen, Oberhausen, Deutschland; 6 Zentrum für Notfallmedizin, BG Klinikum Duisburg, Duisburg, Deutschland; 7 Institut für Medizinische Informatik, Biometrie und Epidemiologie, Universitätsmedizin Essen, Universitätsklinikum Essen, Essen, Deutschland; 8 School of Public Health, Department of Epidemiology, Boston University, Boston, USA; 9 Institut für Virologie, Universitätsmedizin Essen, Universitätsklinikum Essen, Essen, Deutschland; 10 Klinik für Infektiologie, Westdeutsches Zentrum für Infektiologie (WZI), Universitätsmedizin Essen, Universitätsklinikum Essen, Essen, Deutschland

**Keywords:** COVID-19, SARS-CoV‑2, Seroprävalenz, Pandemie, Rettungsdienst, COVID-19, SARS-CoV‑2, Pandemic, Seroprevalence, Emergency medicine

## Abstract

**Hintergrund und Fragestellung:**

Die SARS-CoV-2-Pandemie und die unterschiedliche Ausprägung des Erkrankungsbilds COVID-19 stellen die Gesundheitssysteme weltweit vor eine große Herausforderung. Medizinischem Personal kommt in der Pandemiebekämpfung eine besondere Rolle zu. Ziel der Studie war, die SARS-CoV-2-IgG-Antikörper-Prävalenz bei Personal in der außenklinischen Pandemiebekämpfung in Abhängigkeit von Tätigkeitsbereichen zu untersuchen.

**Methoden:**

Es wurden am 28. und 29.05.2020 von 732 der 1183 Mitarbeitenden (61,9 %) der Berufsfeuerwehr sowie der Hilfsorganisationen im Stadtgebiet Serumproben entnommen und auf SARS-CoV-2-IgG-Antikörper getestet. Entsprechend der Einsatzgebiete wurde das Personal in 4 Kategorien eingeteilt. Kategorie 1: dezentrale PCR-Abstrichteams, Kategorie 2: Rettungsdienst, Kategorie 3: Brandschutz, Kategorie 4: Lagezentrum. Die Tätigkeit des Personals war dabei nicht zwingend auf einen Tätigkeitsbereich beschränkt.

**Ergebnisse:**

In 8 von 732 Serumproben wurden SARS-CoV-2-IgG-Antikörper nachgewiesen. Dies entspricht einer Prävalenz von 1,1 %. Bei 3 Mitarbeitern war eine COVID-19-Infektion schon vor Studienbeginn bekannt. Um eine separate Beurteilung der übrigen Mitarbeiter zu ermöglichen und unbekannte Infektionen zu diagnostizieren, wurde ein korrigiertes Kollektiv aus 729 Mitarbeitern mit 6 SARS-CoV-2-Antikörper-Nachweisen separat betrachtet. Die Prävalenz beträgt im korrigierten Kollektiv 0,82 %. Nach Unterteilung der Kollektive in Tätigkeitsbereiche war die Prävalenz ebenfalls niedrig (1: 0,77 %, 2: 0,9 %, 3: 1,00 %, 4: 1,58 %).

**Schlussfolgerung:**

Die Seroprävalenz von SARS-CoV‑2 im Studienkollektiv ist mit 1,1 % bzw. 0,82 % niedrig. Die Seroprävalenz ist in Tätigkeitsfeldern mit niedriger Gefahr der Virusexposition gegenüber Tätigkeitsfeldern mit größerer Expositionsgefahr erhöht.

**Zusatzmaterial online:**

Die Online-Version dieses Beitrags (10.1007/s10049-021-00948-z) enthält eine Zusammenfassung des Studienkollektivs.

## Abstract (en)

**Background and Objectives:**

The SARS-CoV‑2 pandemic and the different manifestations of the coronavirus disease 2019 (COVID-19) are a major challenge for health systems worldwide. Medical personnel have a special role in containing the pandemic. The aim of the study was to investigate the SARS-CoV‑2 IgG antibody prevalence in extraclinical personnel depending on their operational area in the fight against the COVID-19 pandemic.

**Methods:**

On May 28 and 29, 2020, serum samples were taken from 732 of 1183 employees (61.9%) of the professional fire brigade and aid organizations in the city area and tested for SARS-CoV‑2 IgG antibodies. The employees were divided into four categories according to their type of participation. category 1: decentralized PCR sampling teams, category 2: rescue service, category 3: fire protection, category 4: situation center. Some employees participated in more than one operational area.

**Results:**

SARS-CoV‑2 IgG antibodies were detected in 8 of 732 serum samples. This corresponds to a prevalence of 1.1%. A previous COVID-19 infection was known in 3 employees. In order to make a separate assessment of the other employees possible and to diagnose unknown infections, a corrected collective of 729 employees with 6 SARS-CoV‑2 antibody detection was considered separately. The prevalence in the corrected collective is 0.82%. After subdividing the collective into areas of activity, the prevalence was low (1: 0.77%, 2: 0.9%, 3: 1.00%, 4: 1.58%).

**Conclusions:**

The seroprevalence of SARS-CoV‑2 in the study collective is low at 1.1% and 0.82%, respectively. There is an increased seroprevalence in operational areas with a lower risk of virus exposure in comparison to operational areas with a higher risk.

## Hintergrund und Fragestellung

2019 wurde in Wuhan, China, ein neuartiges Coronavirus identifiziert. Das Virus wurde aufgrund der zuerst erkannten respiratorischen Symptomatik als „severe acute respiratory syndrome coronavirus 2“ (SARS-CoV-2) bezeichnet. Die WHO benannte die zugehörige Erkrankung als „coronavirus disease 2019“ (COVID-19; [23]). Das klinische Bild variiert zwischen asymptomatischen und lebensbedrohlichen Erscheinungsformen [15, 16, 22]. COVID-19 führte zu einer weltweiten Pandemie und besonderen Herausforderung für das Gesundheitssystem [4, 11].

Die internationale Entwicklung der Pandemie führte zu Anpassungsprozessen in der medizinischen Notfallversorgung in Deutschland. Dabei wurden u. a. eine Reduktion des Krankenhausregelbetriebs sowie eine Ausweitung der Intensivbettenkapazitäten und der persönlichen Schutzmaßnahmen des klinischen sowie präklinischen Personals vorgenommen. Regional wurden unter Berücksichtigung der individuellen Infrastruktur unterschiedliche Diagnostik‑, Therapie- sowie Sicherheitskonzepte etabliert. Neben den Anforderungen an persönliche Schutzausrüstungen wurden zentrale und dezentrale Strategien in der Probenentnahme kontrovers diskutiert.

Resultierend wurde für Mitarbeitende des Rettungsdiensts und Brandschutzes eine Anpassung der persönlichen Schutzausrüstung erforderlich. Zur Kontrolle des eigenen Personalschutzkonzepts erfolgt eine Seroprävalenzanalyse nach der 1. Welle der COVID-19-Pandemie. Bei 732 Mitarbeitenden unterschiedlicher Tätigkeitsbereiche wurden semiquantitative ELISA-Tests („enzyme-linked immunosorbent assay“; Fa. EuroImmun Medizinische Labordiagnostika, Lübeck, Deutschland) durchgeführt, sodass die Seroprävalenz standardisiert angegeben und mit einem zuvor untersuchten klinischen Vergleichskollektiv im Stadtgebiet verglichen werden kann [10].

### Lokale Strategie

Bereits vor Beginn der COVID-19-Pandemie wurden in der städtischen Pandemieplanung verschiedene Risiken, wie z. B. Ansteckungsgefahren in Arztpraxen, Kliniken, Diagnostikzentren und in den zuführenden öffentlichen Verkehrsmitteln, definiert. Resultierend wurde in den eigenen Prozessen eine dezentrale Teststrategie festgelegt. Einerseits sollen somit Ansteckungsgefahren reduziert, andererseits auch eine Testverfügbarkeit und Erreichbarkeit für immobile Personen sichergestellt werden.

Die Disposition der dezentralen Testungen erfolgt durch 2 unabhängig arbeitende, jedoch vernetzte, Einsatzleitstellen. Neben der regulär tätigen Einsatzleitstelle der Feuerwehr wird durch abgeordnetes Personal selbiger und Mitarbeitende der unteren Gesundheitsbehörde eine weitere Einsatzleitstelle zur Disposition von Probenentnahmen gebildet.

Medizinische Notfalleinsätze werden unabhängig von einem COVID-19-Verdacht über die Einsatzleitstelle der Feuerwehr disponiert. Der rettungsdienstliche Einsatz erfolgt unter Einhaltung der vorgegebenen Schutzmaßnahmen. Die Probenentnahme erfolgt nach dem Patiententransport entsprechend den innerklinischen Standards durch Klinikpersonal. Die Diagnostik erfolgt in diesen Fällen in einem klinikübergreifend betriebenen Labor oder im Institut für Virologie der Universitätsmedizin.

COVID-19-Verdachtsfälle ohne Hinweise auf einen notfallmedizinischen Behandlungsbedarf werden an eine separate, nur in der Pandemie aktive, Einsatzleitstelle weitergeleitet. In dieser beraten Mitarbeitende der unteren Gesundheitsbehörde, der Feuerwehr und der Universitätsmedizin Anrufer telefonisch. Aufgrund der, insbesondere zu Beginn der Pandemie, oftmals nachvollziehbaren Infektionsketten erfolgt in einer Umfeldanalyse eine detaillierte Aufarbeitung jedes Einzelfalls. Fallabhängig indizieren die Mitarbeiter:innen Probenentnahmen, ordnen Quarantänemaßnahmen an und prüfen Probenergebnisse. Die Disposition der Probenentnahme wird von den abgeordneten Leitstellenmitarbeiter:innen durchgeführt. Die Kooperation der unteren Gesundheitsbehörde und der Feuerwehr wird organisatorisch unter dem Begriff „Lagezentrum der unteren Gesundheitsbehörde“ (LZUGB) geführt. Die Probenentnahmen erfolgen meist in Form von tiefen nasopharyngealen Abstrichen zur Polymerasekettenreaktions(PCR)-Diagnostik. Bei Kleinkindern kann alternativ auch eine Stuhldiagnostik durchgeführt werden. Sämtliche Probenentnahmen werden von Abstrichteams, die aus 2 Personen, Mitarbeitenden der Feuerwehr und Medizinstudierenden, bestehen, durchgeführt. Die anschließende virologische Diagnostik erfolgt im Institut für Virologie der Universität Duisburg-Essen.

Alle Testergebnisse der Kliniken und des LZUGB werden zentral in einer eigenen Datenbank verarbeitet. Bis zum Ende der Untersuchung wurden durch das LZUGB 7322 Befunde der beiden Labore (1254 positiv, 6068 negativ) dokumentiert. 3438 der Proben wurden durch die eigenen Abstrichteams angefertigt. Im Mittel wurden je probennehmender Person ca. 32 Beprobungen durchgeführt. Bei insgesamt 194 SARS-CoV-2-PCR-positiven Personen wurden rettungsdienstliche Notfallbehandlungen (158 RTW, 36 RTW & NEF) durchgeführt. Aufgrund der nicht vorhandenen gesetzlichen Verpflichtung zur Meldung von COVID-19-Negativbefunden wurden nur 396 positive und 461 negative Befunde aus Arztpraxen an das LZUGB übermittelt.

### Tätigkeitsbereiche der Mitarbeiter

Die Mitarbeitenden wurden in den 4 Tätigkeitsbereichen dezentrale Abstrichteams, Rettungsdienst, Brandschutz und LZUGB eingesetzt. Da das Studienkonzept erst Ende April 2020 festgelegt wurde, musste eine retrospektive Zuordnung der Mitarbeiter in verschiedene Tätigkeitsbereiche erfolgen. Mitarbeitende der Hilfsorganisationen waren üblicherweise nur rettungsdienstlich aktiv. Studierende wurden in den Abstrichteams und im LZUGB eingesetzt. Mitarbeitende der Feuerwehr unterstützten in Phasen der Spitzenbelastung alle Tätigkeitsbereiche. Mitarbeitende des Gesundheitsamts waren nur im LZUGB und hatten keinen Patientenkontakt.

### Schutzmaßnahmen

Alle Mitarbeitenden wurden auf die spezifischen Infektionsgefahren in allen Tätigkeitsbereichen, also im Patientenkontakt, in Räumlichkeiten der Feuer- und Rettungswachen, in der Anwendung hygienischer Maßnahmen sowie der Nutzung persönlicher Schutzausrüstung in Abhängigkeit vom Gefährdungspotenzial hingewiesen [21, 22]. Tätigkeiten in Gruppen, wie z. B. im Dienstwechsel des Brandschutzes erforderlich, wurden regelhaft in Außenbereichen durchgeführt. Hospitationen, Angehörigenbegleitungen und Dienstsport wurden während der Pandemiefrühphase in allen Tätigkeitsbereichen vermieden.

Von Personal in den Tätigkeitsbereichen Rettungsdienst und Abstrichteam wurden außerhalb der Fahrzeuge desinfizierende Maßnahmen sowie die Einhaltung von Mindestabständen von 1,50 m erbeten. Im Einsatz und in Einsatzfahrzeugen wurde seit dem 16.03.2020 grundsätzlich das Tragen eines medizinischen Mund-Nasen-Schutzes, der üblichen Einsatzbekleidung und der Gebrauch von Einmalhandschuhen vorgeschrieben. Bei Patientenkontakten mit Verdacht oder Nachweis einer SARS-CoV-2-Infektion wurden ergänzend Schutzbrillen bzw. Schutzvisiere und -kittel angelegt sowie FFP2- oder KN95-Masken (FFP2: partikelfiltrierende Halbmaske, Norm EN 149:2001; KN95: partikelfiltrierende Halbmaske, Norm GB 2626-2006) statt des medizinischen Mund-Nasen-Schutzes getragen (Tab. 1). Erkrankten Patient:innen wurde, falls vertretbar, ebenfalls ein medizinischer Mund-Nasen-Schutz aufgesetzt.

**Table 1:** 

	Rettungsdienst	Brandschutz	Abstrichteam	LZUGB
Außenbereiche	1, 2	1, 2	1, 2	1, 2
Aufenthaltsräume	1, 2	1, 2	1, 2	1, 2
Büroräume (LZUGB)	–	–	–	1
Fahrzeug	2	2	2	–
Patientenkontakt	2	2	–	–
Patientenkontakt (v. a. COVID-19)	3	3	3	–

– = nicht vorhanden, 1 = Abstandsgebot (> 1,50 m) und Reduzierung der Personenanzahl, 2 = Mund-Nasen-Schutz, Einmalhandschuhe, 3 = erweiterte Schutzmaßnahme (FFP2-Maske, Schutzbrille, -visier, -kittel, Einmalhandschuhe)

*LZUGB* Lagezentrum der unteren Gesundheitsbehörde, *FFP* „filtering face piece“, *COVID* „coronavirus disease“

Im Tätigkeitsbereich Brandschutz bestanden identische Vorgaben. Der Kontakt zu Patient:innen entstand jedoch nur im Rahmen einzelner First-Responder-Einsätze. Im Falle von Einsätzen unter Atemschutz wurde auf das Tragen von Mund-Nase-Schutz oder FFP2-Masken verzichtet. Aufgrund des Dienstmodells und der Übergabe in den Außenbereichen bestand die Idee, Kohortierungsmaßnahmen zu erreichen. Diese konnten durch Personalverschiebungen in Spitzenbelastungen nicht immer eingehalten werden.

Auch das LZUGB wurde auf dem Gelände einer Feuerwache eingesetzt. Trotz der räumlichen Separierung waren die Vorgaben der anderen Tätigkeitsbereiche einzuhalten. Den Mitarbeitenden im stets nur administrativen LZUGB wurde, bei gegebener Einhaltung eines Abstands von 1,50 m zum nächstgelegenen Arbeitsplatz, auch in geschlossenen Räumen kein medizinischer Mund-Nasen-Schutz vorgeschrieben. Kontakte zu Patient:innen entstanden nicht.

### Risikobeurteilung

Medizinisches Personal ist aufgrund der besonderen Nähe zu Patient:innen während des Atemwegsmanagements besonders gefährdet [3, 12, 21]. SARS-CoV‑2 zeigt dabei nicht nur eine hohe Infektiosität im Kontaktfall zu erkrankten Personen, sondern im Vergleich zu Influenzaviren auch eine längere Überlebenszeit auf Oberflächen, sodass auch diesbezüglich von einer besonderen Gefährdung des Personals auszugehen ist [3, 8]. In der eigenen Risikobeurteilung stufen wir die Abstrichentnahme aufgrund der vergleichbaren Positionierung des Personals ebenfalls als kritische Maßnahme ein. Auch wenn die Abstrichentnahme planbar ist und nur strukturiert durch ausgebildete Desinfektoren erfolgt, ähnelt das grundsätzliche Infektionsrisiko zeitkritischen rettungsdienstlichen Maßnahmen im Rahmen des Atemwegsmanagements. Resultierend wurde der Einsatz im Rettungsdienst und in den Abstrichteams als Tätigkeit mit hohem Risiko gewertet. Tätigkeiten im Lagezentrum und im Brandschutz sind durch keine bzw. wenige Patientenkontakte gekennzeichnet und weisen somit ein geringeres Infektionsrisiko auf. Dieses ist mit dem der Allgemeinbevölkerung vergleichbar.

Aufgrund des Gefährdungspotenzials ist eine Erhebung der SARS-CoV-2-IgG-Antikörper-Seroprävalenz in der Mitarbeiterschaft, adaptiert nach Tätigkeitsbereichen, als Qualitätssicherungsmaßnahme der eingeleiteten Schutzmaßnahmen im Rahmen der Pandemiebekämpfung zu verstehen.

## Studiendesign und Untersuchungsmethoden

Untersucht wurden insgesamt 732 von 1183 Mitarbeitenden (61,9 %) aus (1) Rettungsdienst, (2) Abstrichteams, (3) Brandschutz und (4) LZUGB, die entsprechend ihrer Tätigkeitsbereiche kategorisiert wurden. Mehrfachnennungen der Tätigkeitsbereiche waren möglich.

Aufgrund des Dienstplanmodells der Feuerwehr waren 3 Untersuchungstage erforderlich, um allen Mitarbeitenden eine Studienteilnahme zu ermöglichen. Die Blutentnahmen wurden intern vorangekündigt und im Zeitraum vom 27. bis 29. Mai 2020 durchgeführt. Auch Mitarbeitende im 24-Stunden-Dienstmodell konnten jeweils an mindestens 2 Terminen zur Blutentnahme erscheinen. Den Untersuchern sind keine Fälle bekannt, in denen Mitarbeitende aufgrund von Quarantänemaßnahmen keine Möglichkeit zur Teilnahme hatten. Seitens der Arbeitgeber wurde auch innerhalb der Arbeitszeit eine Vorstellung ermöglicht. Eine vorherige Terminierung war nicht erforderlich. Die Mitarbeiter wurden über die Studie und Blutentnahme aufgeklärt. Eine Teilnahme war freiwillig.

Nach der Blutentnahme folgten eine unmittelbare Kühlung, Lagerung sowie ein Transport in das virologische Institut der Universität Duisburg-Essen am Folgetag. Die Serumproben wurden in den beiden Folgewochen in semiquantitativen ELISA-Tests (Fa. EuroImmun Medizinische Labordiagnostika, Lübeck, Deutschland) auf Anti-SARS-CoV-2-IgG-Antikörper geprüft.

Die Sensitivität liegt in Woche 1 nach positivem PCR-Befund bei 21,6 %, in Woche 2 bei 55,1 % und in Woche 3 bei 89,5 % [19]. Die mediane Serokonversion wurde bei erkrankten Personen nach 10,7 Tagen (95 % KI 9,6–11,9) beschrieben [9]. In Mischkollektiven wurde die Sensitivität ab dem 14. Tag nach Symptombeginn bei bestehender positiver PCR-Testung mit 100 % angegeben [13, 14]. Das Absinken der Seroprävalenz ist jedoch inhomogen. Ein Absinken der Serumspiegel wurde in Patientenkollektiven ab dem 4. Monat festgestellt. Die mediane Nachweisdauer liegt bei 168,5 Tagen (62–199 Tage; [5, 18, 20]). Die Spezifität der Anti-SARS-CoV-2-ELISA-Testung für IgG-Antikörper wurde zwischen 91,5 und 95,8 % angegeben [1, 13, 14].

## Statistische Methoden

Die Studie wurde als retrospektive Beobachtungsstudie durchgeführt. Die Berechnung der demografischen Angaben erfolgte in Microsoft Excel für Mac (Version 16.48) und SAS Statistical Analysis Software (Version 9.4). Die Angaben der normalverteilten Werte erfolgt als Median und Interquartilsabstand (IQR). Kategoriale Daten werden als Anzahl (*n*) und Anteil (%) dargestellt.

Ergänzt wurden anschließend bundeslandbezogene Angaben zu SARS-CoV-2-Infektionen und die assoziierten Todesfälle in Deutschland bis zum 23.02.2021 aus dem COVID-19-Data-Hub. In diesem vereinigt und präsentiert das Robert Koch-Institut (RKI) bundesweite Daten des pandemischen Geschehens und stellt diese in konsolidierter Form zur Verfügung [17]. Um den zeitlichen Zusammenhang der Seroprävalenzanalyse auf die regionalen Pandemiephasen zu ermöglichen, erfolgt eine Addition der täglichen Einzelmeldungen der meldenden Landkreise und Städte sowie anschließend eine Glättung der Zeitreihen mittels „simple moving average“ (SMA = gleitender Durchschnitt) der Periode 7 Tage. Dargestellt sind neben den SARS-CoV-2-Neuinfektionen und -assoziierten Todesfällen die Untersuchungsintervalle der in Deutschland durchgeführten Studien.

Die Studie wurde vom Ethikkomitee der Medizinischen Fakultät der Universität Duisburg-Essen geprüft und die Durchführung bewilligt (20-9208-BO).

## Ergebnisse

**Figure 1:**
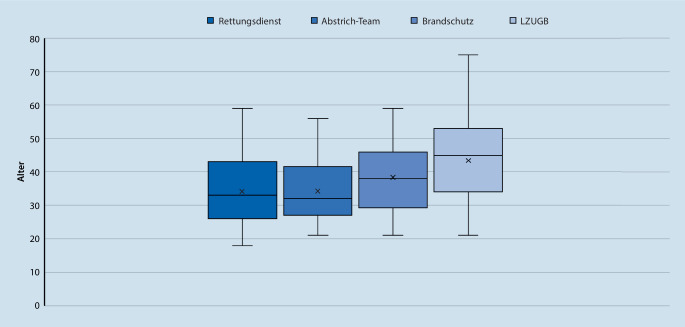

Insgesamt 732 von 1183 Mitarbeitenden (61,9 %) erklärten sich nach Bekanntgabe der anstehenden Studie zur Teilnahme bereit. Die Mitarbeitenden waren häufiger männlichen Geschlechts (Tab. 2). Das Personal in den risikobehafteten Tätigkeitsbereichen Rettungsdienst und Abstrichteams waren dabei tendenziell jünger als die Mitarbeitenden in Brandschutz und LZUGB (Abb. 1).

**Table 2:** 

	Gesamtkollektiv				Männlich				Weiblich			
	Gesamt	+	−	SP (in %)	Gesamt	+	−	SP (in %)	Gesamt	+	−	SP (in %)
Alle	732	8	724	1,09	617	6	611	0,97	115	2	113	1,74
Abstrich	130	1	129	0,77	126	1	125	0,79	4	0	4	0,00
RD	441	4	437	0,91	406	4	402	0,99	35	0	35	0,00
Brandschutz	397	4	393	1,01	391	4	387	1,02	6	0	6	0,00
LZUGB	253	4	249	1,58	177	2	175	1,13	76	2	74	2,63

*RD* Rettungsdienst, *SP* Seroprävalenz, *LZUGB* Lagezentrum der unteren Gesundheitsbehörde

Bei 8 der 732 (1,09 %) Personen konnten in der ELISA-Diagnostik SARS-CoV-2-IgG-Antikörper nachgewiesen werden (Tab. 2). 3 Studienteilnehmer wurden im Vorfeld der Studie reiseassoziiert SARS-CoV-2-positiv getestet. Sie beschrieben symptomatische Verläufe unterschiedlicher Ausprägung. Bei 2 dieser Teilnehmer konnten in der ELISA-Diagnostik IgG-Antikörper nachgewiesen werden. Im korrigierten Kollektiv ohne vorbekannte SARS-CoV-2-Infektionen erfolgte somit bei 6 von 729 (0,82 %) ein SARS-CoV-2-IgG-Antikörper-Nachweis. Die Tätigkeitsbereiche der positiv getesteten Mitarbeitenden sind in Tab. 3, die des gesamten Studienkollektivs nur in der digitalen Version des Artikels dargestellt (Online-Zusatzmaterial, siehe Box am Anfang).

**Table 3:** 

ID	Alter	Geschlecht	Rettungsdienst	Brandschutz	Abstrichteam	Lagezentrum
17100019	51	M	+	+	+	−
*17100066*	*53*	*M*	*+*	*+*	*−*	*+*
17100102	35	W	−	−	−	+
17100387	46	M	+	+	−	−
17100395	38	M	+	−	−	−
*17100420*	*31*	*M*	*−*	*−*	*−*	*+*
17100458	22	W	−	−	−	+
17100634	52	M	−	+	−	−

Vorbekannte Infektionen werden kursiv dargestellt

+ = ja, − = nein

Während die Infektionswege der im Vorfeld positiven Fälle aufgrund der Reiseanamnese mit anschließender Symptomatik bekannt waren, konnten die Infektionsquellen der übrigen positiv getesteten Studienteilnehmenden nicht eruiert werden. Die Seroprävalenzanalyse der Tätigkeitsbereiche ist jeweils niedrig (Tab. 2). In den als Hochrisikobereiche definierten Rettungsdienst- (1) und Abstrichteams (2) sind die Seroprävalenzen niedriger als im Brandschutz (3) und im LZUGB (4) (1: 0,91 %, 2: 0,77 %, 3: 1,01 %, 4: 1,58 %). Die Seroprävalenz von Personen, die nur in den risikoreichen Tätigkeitsbereichen Rettungsdienst und Abstrichteams, nicht jedoch in LZUGB und Brandschutz tätig waren (*n* = 136) lag bei 0,7 %. Die Seroprävalenz von Personen, die nur in LZUGB und Brandschutz, nicht jedoch in Rettungsdienst und Abstrichteams eingesetzt wurden (*n* = 276), betrug 1,1 %.

Es kam nicht zu gehäuften Positivbefunden in einzelnen Feuer- bzw. Rettungswachen. Keine der teilnehmenden Personen befand sich aufgrund einer SARS-CoV-2-Infektion in stationärer medizinischer Behandlung.

## Diskussion

Ziel unserer Studie war die Kontrolle der Seroprävalenz bei den Mitarbeitenden der unteren Gesundheitsbehörde, der Feuerwehr und Hilfsorganisationen in der außerklinischen Bekämpfung der COVID-19-Pandemie. Verglichen wurden Personen unterschiedlicher Tätigkeitsbereiche, die in eine Gruppe mit Patientenkontakten und attestiertem höherem Risiko sowie eine Gruppe ohne Patientenkontakte und mit angenommenem geringerem Risiko eingeteilt wurden.

Nationale Studien mit isolierter Betrachtung des außerklinischen Personals in der Pandemiebekämpfung sind bisher nicht bekannt. Zwar wurden in einigen Studien Seroprävalenzen auch von außerklinischem Rettungsdienstpersonal bestimmt, jedoch fand sich das Rettungsdienstpersonal – sicherlich auch aufgrund unterschiedlicher notfallmedizinischer Konzepte – in einem Kollektiv mit Mitarbeitenden der Notaufnahmen und Pflegepersonal wieder. In der eigenen Studie gelingt ebenfalls keine isolierte Darstellung des Rettungsdienstpersonals, jedoch kann das Risiko des Personals in der außerklinischen Pandemiebekämpfung dargestellt werden. Aufgrund der fehlenden Trennschärfe erfolgt in der Diskussion eine vergleichende Darstellung der Seroprävalenzstudien von Gesundheitsberufen mit vergleichbaren Risikoprofilen.

**Figure 2:**
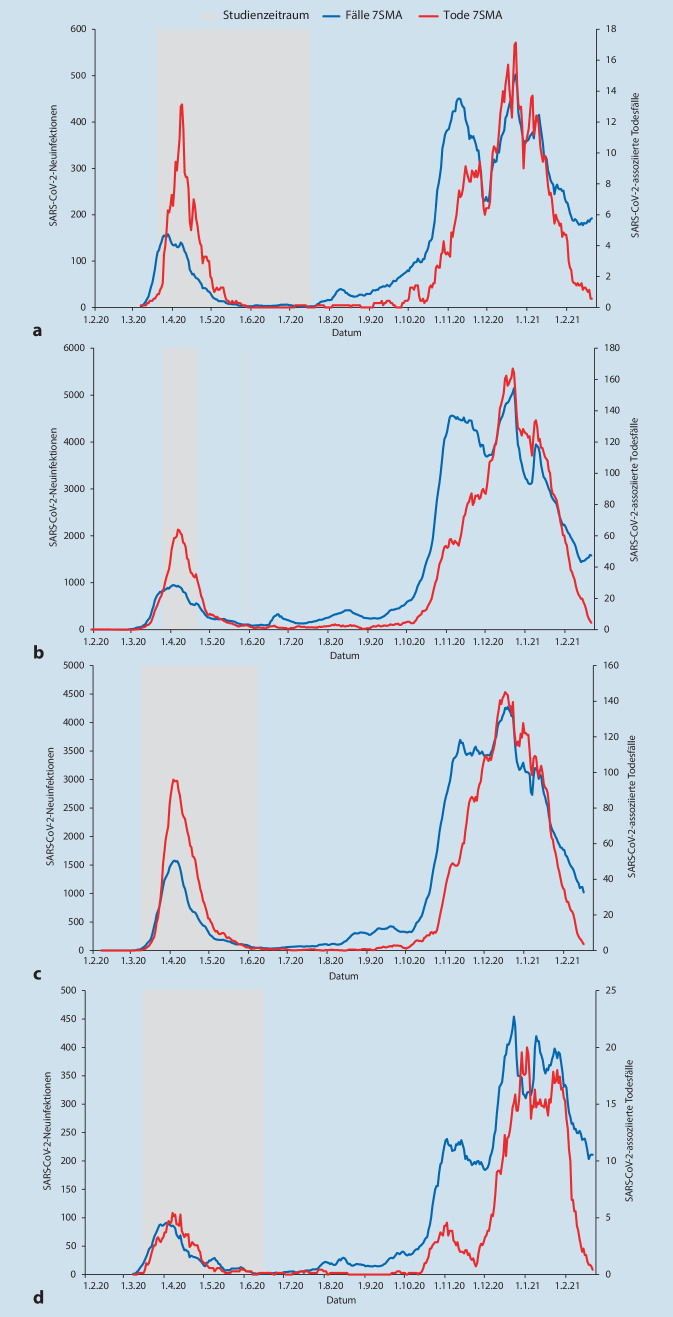

In der Betrachtung der Seroprävalenzen sind dabei verschiedene Einflussfaktoren zu berücksichtigen. Neben der beruflichen Tätigkeit ist dies vor allem die Expositionswahrscheinlichkeit, die im Pandemieverlauf durch die regionalen Neuinfektions- und Todesfallzahlen sowie die Bevölkerungsdichte bestimmt wird (Abb. 2). Eine vom Zeitpunkt der Pandemie abhängige Betrachtung der Seroprävalenzen erscheint insbesondere für das Kollektiv mit Patientenkontakt relevant. So können Todesfallzahlen als Hinweis auf eine rettungsdienstliche Tätigkeit in zugehörigen medizinischen Notfällen verstanden werden. In der eigenen Strategie ist auch die Anzahl der Neuinfektionen als Hinweis auf die Expositionswahrscheinlichkeit der Abstrichteams bedeutend.

In einer Vorstudie im eigenen Stadtgebiet wurde in Hochrisikotätigkeitsbereichen eine niedrigere Seroprävalenz als in den Bereichen mit niedrigem Risiko festgestellt [10]. Korth et al. führten bei einer eindeutigen Zuordnung der Tätigkeitsbereiche die niedrigere Seroprävalenz in Hochrisikotätigkeitsbereichen auf den erfahreneren Umgang mit persönlicher Schutzausrüstung zurück. Zwar wurde in der Datenauswertung der eigenen Studie der Personaleinsatz zahlreicher Mitarbeitender aus Hochrisikobereichen auch in Tätigkeitsbereichen mit niedrigem Risiko festgestellt, jedoch verbleiben Personen, die nur in Hoch- oder Niedrigrisikobereichen eingesetzt wurden (Tab. 2 und 4, Online-Zusatzmaterial). Die Seroprävalenz von Personen, die nur in den risikoreichen Tätigkeitsbereichen waren, lag mit 0,7 % unter der Gesamtseroprävalenz. Die Seroprävalenz von Personen, die nur in Tätigkeitsbereichen mit niedrigem Risiko tätig waren, lag bei 1,1 % und war somit deutlich höher als die des Gesamtkollektivs. Das Personalschutzkonzept der Hochrisikotätigkeitsfelder kann somit als suffizient eingestuft werden.

**Table 4:** 

	Anzahl	Rettungsdienst	Brandschutz	Abstrichteam	LZUGB
Rettungsdienst	441	–	302	121	31
Brandschutz	397	302	–	123	59
Abstrichteams	130	121	123	–	11
LZUGB	253	31	59	11	–

*LZUGB* Lagezentrum der unteren Gesundheitsbehörde

Im weiteren Verlauf werden, aufgrund der fehlenden Trennschärfe, vereinfachend die Daten des Gesamtkollektivs diskutiert. Die Vergleiche sind stets unter Berücksichtigung der Expositionswahrscheinlichkeit zu verstehen und stets in zeitlichen und regionalen Bezug zu Pandemiephasen zu setzen.

Im Vergleich zu einer frühen Beschreibung von SARS-CoV-2-Antikörpern bei medizinischem Personal durch Chen et al. sind die eigenen Ergebnisse als Folge eines suffizienten Schutzkonzepts zu verstehen. Im chinesischen Kollektiv in der Provinz Nanjing wurde bereits in der Frühphase der 1. Welle zwischen dem 12. und dem 20.02.2020 bei 105 Mitarbeitenden des Gesundheitsdiensts, etwa 500 km Luftlinie von Wuhan entfernt, mit Kontakt zu 4 COVID-19-Patienten eine Seroprävalenz von 17,14 % diagnostiziert [3]. Kenntnisse über die erforderlichen Schutzmaßnahmen und die technischen Voraussetzungen zur Sicherstellung einer flächendeckenden labormedizinischen Diagnostik waren zu diesem Zeitpunkt jedoch noch nicht gesichert.

Da in internationalen Publikationen sowohl der Vergleich der notfallmedizinischen Konzepte und der regionalen Pandemiephasen als auch die Art und Verfügbarkeit von labormedizinischer Diagnostik einen Vergleich von Seroprävalenzen erschwert, werden im Folgenden die nationalen Publikationen zu Seroprävalenzen in notfallmedizinischen bzw. -nahen Kollektiven diskutiert. Eine vergleichbare Verfügbarkeit der labormedizinischen Diagnostik in Studien wird hierbei angenommen. In den meisten der im Verlauf diskutierten Studien wurde, wie auch in der eigenen Studie, der EuroImmun-Anti-SARS-CoV-2-ELISA-Test genutzt und eine Beurteilung der IgG-Antikörper durchgeführt.

Erste nationale Seroprävalenzanalysen erfolgten in etwa zeitgleich in 2 Universitätskliniken in Essen und Hamburg (Abb. 2a,b). Während in Hamburg 3 sequenzielle Untersuchungen im Sinne einer Periodenprävalenz zwischen dem 20. März und 17. Juli 2020 bei 1253 Mitarbeitenden vorgenommen wurden, erfolgte in Essen eine einmalige Untersuchung von 316 Personen (Abb. 2a; 1. Zeitraum; [2, 10]). Im Hamburger Kollektiv waren 1026 der Mitarbeitenden in der Patientenversorgung tätig. Zum Ende der Studie gelang bei insgesamt 22 Personen (1,8 %) ein Antikörpernachweis, was für eine suffiziente Infektprävention am Arbeitsplatz spricht [2]. In der Essener Studie wurden bei 5 Mitarbeitenden (1,6 %) Antikörper nachgewiesen. Die Interpretation der Essener Arbeitsgruppe sieht bei wenigen Antikörpernachweisen keine Häufung in Hochrisikoarbeitsbereichen. Beide Studien wurden jedoch bereits innerhalb der 1. Welle der COVID-19-Pandemie durchgeführt, wodurch möglicherweise Untersuchungen bereits vor Serokonversion durchgeführt worden sein könnten.

Der eigenen Einschätzung nach ist die in der eigenen Untersuchung gewählte Punktprävalenzanalyse besser auf eine vorherige Pandemiephase zu beziehen als eine, den Beginn der Pandemie einschließende, Periodenprävalenz. Voraussetzung ist, dass die Punktprävalenzanalyse in einem günstigen Zeitabstand zum Bezugsintervall erfolgt.

Die Ergebnisse der Essener Arbeitsgruppe sind aufgrund der regionalen und zeitlichen Nähe, ca. 4–8 Wochen vor der eigenen Untersuchung, sowie der Durchführung im selben virologischen Institut gut mit den eigenen Studienergebnissen vergleichbar. Auffällig ist hierbei die, trotz der späteren Untersuchung, niedrigere Prävalenz im eigenen Kollektiv. Da der Zeitabstand der Untersuchung zur 1. Welle der COVID-19-Pandemie länger als die Serokonversionsdauer ist, kann das eigene Schutzkonzept als erfolgreich bewertet werden.

Weitere publizierte deutsche Screeninguntersuchungen mit notfallmedizinischen Kollektiven wurden im Hamburger Umland durchgeführt (Abb. 2c). In regionaler Nähe zu Hamburg untersuchten Herzberg et al. ebenfalls in Form einer Periodenprävalenz mit ähnlichem Zeitraum in einer Screeninguntersuchung 871 von 1081 Klinikmitarbeitern [7]. Bei 38 Studienteilnehmenden (4,36 %) wurden SARS-CoV-2-IgG-Antikörper nachgewiesen. Somit liegt in regionaler Nähe bei in etwa zeitgleichem Studienbeginn eine deutlich höhere Prävalenz als zuvor in Hamburg vor [2].

Im Vergleich zum eigenen Kollektiv wurden die Analysen in beiden Studien bereits in der 1. Welle der COVID-19-Pandemie durchgeführt. Die absoluten Neuinfektions- und Todesfallzahlen sprechen bezogen auf die Bevölkerungszahl jedoch auch für eine größere Belastung des Gesundheitssystems von Hamburg im Vergleich zu NRW, wodurch eine höhere Seroprävalenz in der Allgemeinbevölkerung ebenfalls erklärt werden kann. Aufgrund der diskutierten Serokonversionsdauer und Antikörperpersistenz erscheint ein weiterer Anstieg bis zu einem späteren, zur eigenen Studie vergleichbaren, Zeitpunkt wahrscheinlich.

Regional unabhängig von den zuvor diskutierten Daten publizierten Finkenzeller et al. im Sommer 2020 in Bayern erhobene Daten (Abb. 2d; [6]). Die Autoren wählten einen größeren Zeitabstand zur 1. Welle der COVID-19-Pandemie, sodass – wie auch im eigenen Studienkonzept – eine Serokonversion des Kollektivs hochwahrscheinlich ist. Vom 29. Juni zum 29. Juli 2020 wurden 1838 von 2387 Krankenhausmitarbeitenden (77,0 %) und 986 von 1850 Mitarbeitenden eines Vergleichskollektivs ohne Patientenkontakt (53,3 %) untersucht. Insgesamt wurden bei 313 Personen (11,1 %) SARS-CoV-2-IgG-Antikörper nachgewiesen. Die Seroprävalenz lag in der Gruppe mit Patientenkontakt bei 15,1 %, in der Gruppe ohne Patientenkontakt bei 3,6 %. Sowohl die Neuinfektions- als auch die Todesfallzahlen waren dabei in Bayern, bezogen auf die Bevölkerungszahl, deutlich höher als in NRW.

Während die eigene Studie die Verlaufsdarstellung zum Kollektiv mit beruflicher Tätigkeit innerhalb eines Stadtgebiets ermöglicht, sind in den anderen Fällen Vergleiche von Kliniken in Stadtlage mit ländlichen bis suburbanen Studienregionen erforderlich. In Folge ist in der Beurteilung der eigenen Strategie der fehlende Anstieg der Seroprävalenz im regional unterschiedlichen Verlauf nach der 1. Welle der COVID-19-Pandemie als Erfolg zu verstehen.

Der Vergleich innerhalb derselben Region betont im eigenen Verständnis das schlüssige Infektionsschutzkonzept für die eigenen Mitarbeiter unter der Annahme, dass eine Serokonversion wenige Tage bis Wochen nach Viruskontakt erfolgt und anschließend über mehrere Monate ein Antikörpernachweis möglich ist.

## Limitationen

Um die Aussagekraft der Studie zu erhöhen, wäre eine eindeutige Zuordnung der Mitarbeitenden durch eine geringere Überschneidung der Tätigkeitsbereiche wünschenswert. Ebenso könnte die Qualität der Hygienekonzepte in der 2. und 3. Welle der COVID-19-Pandemie mit höheren Inzidenzen und Einsatzzahlen im Rettungsdienst besser beurteilt werden. Dies ist aufgrund der begonnenen Impfmaßnahmen nicht mehr möglich. Zuletzt kann aufgrund des Studienzeitpunkts keine Aussage über die Seroprävalenzentwicklung nach Auftreten von Virusmutationen getroffen werden.

## Schlussfolgerungen

Die Seroprävalenz im untersuchten Studienkollektiv ist unter Berücksichtigung des Untersuchungszeitpunkts und des regionalen Verlaufs der COVID-19-Pandemie vergleichsweise niedrig. Zwar zeigen sich deutliche Überschneidungen der Tätigkeitsbereiche des Personals, jedoch bestand in Subkollektiven mit niedriger beruflicher Expositionswahrscheinlichkeit eine im Vergleich höhere SARS-CoV-2-Antikörper-Seroprävalenz als im Subkollektiv mit einer höheren Expositionswahrscheinlichkeit. Häufigere Kontakte zu Patient:innen mit schweren Krankheitsverläufen und resultierend ein erfahrener und bewussterer Umgang mit Schutzmaßnahmen können Hintergrund dieser Verteilung sein. Da nicht nachvollzogen werden kann, ob die Infektion während der beruflichen Tätigkeit oder im privaten Umfeld erfolgte, ist die Schärfung des Bewusstseins zu einem konsequenten Einhalten von Schutzmaßnahmen im privaten sowie beruflichen Alltag erforderlich. Eine Anpassung der persönlichen Schutzausrüstung für die Hochrisikotätigkeitsbereiche, die im Regelfall aus der Dienstbekleidung, einem medizinischen Mund-Nasen-Schutz sowie Handschuhen und im Verdachtsfall ergänzend aus einer Schutzbrille bzw. Schutzvisier und -kittel sowie einer FFP2-/KN95-Maske statt einem medizinischen Mund-Nasen-Schutz besteht, scheint nicht erforderlich.

## Supplementary Information




