# Supplementary material for: SARS-CoV-2 IgG seroprevalence in personnel of the extraclinical fight against the COVID-19 pandemic
Source: Notf Rett Med. 2021 Oct 12:1–9. [Article in German] Online ahead of print. doi: 10.1007/s10049-021-00948-z (PMC8507503; doi:10.1007/s10049-021-00948-z)
Supplement: Supplementary file 1 [file 10049_2021_948_MOESM1_ESM.pdf]

Zusatzmaterial zum Beitrag „SARS-CoV-2-IgG-Antikörperseroprävalenz bei Personal in der außerklinischen Bekämpfung der COVID-19-Pandemie“ von Brune B, Koth J, Fessmann K et al. (2021) in *Notfall+Rettungsmedizin* .

Beitrag und Zusatzmaterial stehen Ihnen auf www.springermedizin.de zur Verfügung. Bitte geben Sie dort den Beitragstitel in die Suche ein.

Studien-nummer	Alter	Geschlecht	Rettungs-dienst	Abstrich-Team	Brandschutz	Lage-zentrum	CLIA-Ergebnis neg/pos
17100019	51	m	ja	ja	ja	nein	positiv
17100066	53	m	ja	nein	ja	ja	positiv
17100102	35	w	nein	nein	nein	ja	positiv
17100387	46	m	ja	nein	ja	nein	positiv
17100395	38	m	ja	nein	nein	nein	positiv
17100420	31	m	nein	nein	nein	ja	positiv
17100458	22	w	nein	nein	nein	ja	positiv
17100634	52	m	nein	nein	ja	nein	positiv
17100001	42	w	nein	nein	nein	ja	negativ
17100002	43	m	ja	ja	ja	nein	negativ
17100003	47	m	ja	nein	ja	nein	negativ
17100004	26	w	ja	ja	ja	nein	negativ
17100005	33	m	ja	nein	ja	nein	negativ
17100006	52	m	nein	nein	nein	ja	negativ
17100007	48	m	nein	nein	ja	ja	negativ
17100008	57	m	ja	nein	ja	nein	negativ
17100010	37	m	ja	nein	ja	nein	negativ
17100011	52	m	nein	nein	nein	ja	negativ
17100012	32	m	nein	nein	ja	nein	negativ
17100013	60	m	nein	nein	nein	ja	negativ
17100014	30	m	nein	nein	ja	nein	negativ
17100015	55	w	nein	nein	nein	ja	negativ
17100016	52	m	nein	nein	ja	nein	negativ
17100017	32	m	ja	nein	ja	nein	negativ
17100018	43	m	nein	nein	nein	ja	negativ
17100020	55	m	nein	nein	nein	ja	negativ
17100021	29	m	ja	nein	ja	nein	negativ
17100022	50	m	nein	nein	ja	nein	negativ
17100023	42	w	nein	nein	ja	ja	negativ
17100024	54	m	nein	nein	ja	nein	negativ
17100025	51	m	ja	nein	ja	nein	negativ
17100026	40	m	ja	nein	ja	nein	negativ
17100027	35	m	ja	nein	ja	nein	negativ
17100028	55	m	nein	nein	ja	nein	negativ
17100029	43	m	nein	nein	ja	ja	negativ
17100030	34	m	nein	nein	ja	nein	negativ
17100031	50	w	nein	nein	nein	ja	negativ
17100032	46	m	ja	ja	ja	nein	negativ
17100033	53	m	nein	nein	nein	ja	negativ

17100034	47	w	nein	nein	nein	ja	negativ
17100035	40	m	ja	nein	ja	nein	negativ
17100036	56	m	nein	nein	ja	nein	negativ
17100037	40	m	ja	ja	ja	ja	negativ
17100038	59	m	ja	nein	nein	nein	negativ
17100039	52	m	ja	nein	ja	nein	negativ
17100040	26	m	ja	nein	ja	nein	negativ
17100041	55	m	nein	nein	ja	nein	negativ
17100042	43	m	nein	nein	nein	ja	negativ
17100043	43	m	ja	nein	ja	nein	negativ
17100044	44	m	ja	ja	ja	nein	negativ
17100045	24	m	ja	ja	ja	nein	negativ
17100046	30	w	nein	nein	nein	ja	negativ
17100047	54	m	nein	nein	nein	ja	negativ
17100048	33	w	nein	nein	nein	ja	negativ
17100049	56	w	nein	nein	nein	ja	negativ
17100050	44	m	nein	nein	ja	nein	negativ
17100051	59	w	nein	nein	nein	ja	negativ
17100052	31	m	ja	nein	ja	nein	negativ
17100053	46	m	nein	nein	nein	ja	negativ
17100054	33	m	ja	ja	ja	nein	negativ
17100055	54	m	nein	nein	nein	ja	negativ
17100056	49	m	ja	nein	nein	ja	negativ
17100057	50	m	nein	nein	nein	ja	negativ
17100058	28	w	nein	nein	nein	nein	negativ
17100059	59	m	nein	nein	nein	ja	negativ
17100060	52	m	nein	nein	nein	ja	negativ
17100061	28	m	ja	nein	ja	nein	negativ
17100062	41	m	nein	nein	ja	nein	negativ
17100063	28	m	ja	nein	ja	nein	negativ
17100064	23	m	ja	ja	ja	nein	negativ
17100065	24	m	ja	ja	ja	nein	negativ
17100067	47	m	ja	nein	ja	nein	negativ
17100068	41	m	nein	nein	nein	ja	negativ
17100069	36	m	ja	ja	ja	nein	negativ
17100070	45	m	ja	nein	ja	ja	negativ
17100071	42	m	ja	nein	nein	ja	negativ
17100072	28	m	ja	ja	ja	nein	negativ
17100073	28	m	ja	ja	ja	nein	negativ
17100074	67	m	nein	nein	nein	ja	negativ
17100075	50	m	nein	nein	ja	ja	negativ
17100076	41	m	ja	nein	ja	nein	negativ
17100077	53	m	ja	nein	ja	nein	negativ
17100078	39	m	ja	ja	ja	nein	negativ
17100079	43	m	ja	nein	ja	nein	negativ
17100080	38	m	nein	nein	nein	ja	negativ
17100081	32	m	nein	nein	ja	ja	negativ
17100082	47	m	nein	nein	nein	ja	negativ
17100083	50	m	ja	nein	ja	nein	negativ
17100084	45	m	ja	ja	ja	nein	negativ

17100085	44	m	nein	nein	nein	ja	negativ
17100086	55	m	nein	nein	nein	ja	negativ
17100088	39	m	nein	nein	nein	ja	negativ
17100089	51	m	nein	ja	nein	ja	negativ
17100090	41	m	ja	nein	ja	nein	negativ
17100091	58	m	nein	nein	ja	ja	negativ
17100092	30	m	nein	nein	nein	ja	negativ
17100093	56	m	nein	nein	nein	ja	negativ
17100094	45	m	ja	nein	ja	nein	negativ
17100095	47	m	nein	nein	nein	ja	negativ
17100096	45	m	nein	nein	nein	ja	negativ
17100097	54	w	nein	nein	nein	ja	negativ
17100098	39	m	ja	nein	ja	nein	negativ
17100099	33	m	ja	nein	ja	ja	negativ
17100100	28	m	nein	nein	nein	ja	negativ
17100101	40	m	nein	ja	ja	nein	negativ
17100103	29	m	ja	nein	ja	nein	negativ
17100104	21	m	ja	ja	ja	nein	negativ
17100105	53	m	ja	nein	ja	nein	negativ
17100106	42	m	nein	nein	ja	ja	negativ
17100107	51	m	nein	nein	nein	ja	negativ
17100108	42	m	nein	nein	nein	ja	negativ
17100109	49	m	nein	ja	nein	nein	negativ
17100110	51	m	nein	nein	nein	ja	negativ
17100111	43	m	ja	nein	ja	nein	negativ
17100112	46	m	nein	nein	nein	ja	negativ
17100113	32	m	nein	ja	nein	ja	negativ
17100114	27	m	ja	ja	ja	nein	negativ
17100115	34	m	ja	nein	ja	nein	negativ
17100116	52	m	nein	nein	nein	ja	negativ
17100117	34	m	ja	nein	ja	ja	negativ
17100118	30	m	ja	ja	ja	nein	negativ
17100119	24	m	ja	ja	ja	nein	negativ
17100120	52	m	ja	nein	ja	nein	negativ
17100121	41	m	ja	ja	ja	nein	negativ
17100122	37	m	nein	nein	ja	ja	negativ
17100123	30	m	ja	ja	ja	nein	negativ
17100124	57	m	nein	nein	ja	nein	negativ
17100125	24	m	ja	ja	ja	ja	negativ
17100126	59	w	nein	nein	nein	ja	negativ
17100127	52	m	ja	nein	ja	ja	negativ
17100128	53	m	nein	nein	ja	ja	negativ
17100129	55	m	nein	nein	nein	ja	negativ
17100130	28	m	ja	ja	ja	nein	negativ
17100131	52	m	ja	ja	ja	nein	negativ
17100132	48	m	ja	nein	ja	nein	negativ
17100133	41	m	nein	nein	ja	ja	negativ
17100134	50	m	ja	nein	nein	nein	negativ
17100135	41	m	ja	nein	ja	nein	negativ
17100136	59	m	nein	nein	nein	ja	negativ

17100137	60	m	nein	nein	nein	ja	negativ
17100138	39	m	nein	nein	ja	nein	negativ
17100139	47	w	nein	nein	nein	ja	negativ
17100140	56	m	ja	nein	ja	nein	negativ
17100143	43	m	nein	nein	ja	ja	negativ
17100144	58	m	nein	nein	ja	nein	negativ
17100145	45	m	nein	nein	ja	ja	negativ
17100146	24	m	ja	nein	ja	nein	negativ
17100147	39	m	nein	nein	ja	nein	negativ
17100148	42	m	nein	nein	nein	ja	negativ
17100149	34	m	ja	ja	ja	nein	negativ
17100150	43	m	nein	nein	ja	nein	negativ
17100151	28	m	ja	ja	ja	nein	negativ
17100152	36	m	ja	nein	ja	nein	negativ
17100153	33	m	nein	nein	ja	nein	negativ
17100154	32	m	ja	ja	ja	nein	negativ
17100155	57	m	nein	nein	nein	ja	negativ
17100156	56	m	nein	nein	ja	nein	negativ
17100157	41	m	nein	nein	nein	ja	negativ
17100158	60	w	nein	nein	nein	ja	negativ
17100160	49	m	ja	nein	ja	nein	negativ
17100161	35	m	ja	nein	ja	nein	negativ
17100162	30	m	ja	nein	ja	nein	negativ
17100163	42	m	ja	nein	ja	nein	negativ
17100164	48	w	nein	nein	nein	ja	negativ
17100165	54	m	nein	nein	ja	nein	negativ
17100166	49	m	nein	nein	nein	ja	negativ
17100167	49	m	nein	nein	ja	ja	negativ
17100168	49	m	ja	ja	ja	nein	negativ
17100169	33	m	ja	nein	ja	ja	negativ
17100170	55	m	nein	nein	nein	ja	negativ
17100171	35	m	ja	nein	ja	nein	negativ
17100172	35	w	ja	ja	ja	nein	negativ
17100173	34	m	nein	nein	nein	ja	negativ
17100174	29	m	ja	ja	ja	nein	negativ
17100175	51	m	nein	nein	nein	ja	negativ
17100176	29	m	ja	nein	ja	nein	negativ
17100177	57	m	nein	nein	nein	ja	negativ
17100178	47	m	ja	ja	ja	nein	negativ
17100179	23	m	ja	ja	ja	nein	negativ
17100180	46	w	nein	nein	ja	ja	negativ
17100181	59	w	nein	nein	nein	ja	negativ
17100182	47	m	ja	nein	ja	nein	negativ
17100183	45	m	nein	nein	ja	ja	negativ
17100184	57	m	nein	nein	ja	ja	negativ
17100185	52	m	ja	nein	ja	ja	negativ
17100186	51	m	nein	nein	nein	ja	negativ
17100187	35	m	ja	nein	ja	ja	negativ
17100188	52	m	ja	nein	ja	ja	negativ
17100189	38	m	ja	nein	ja	nein	negativ

17100190	51	m	ja	nein	ja	nein	negativ
17100191	37	m	nein	nein	nein	ja	negativ
17100192	40	m	ja	nein	ja	nein	negativ
17100193	54	m	nein	nein	ja	ja	negativ
17100194	26	m	ja	nein	ja	nein	negativ
17100195	39	m	ja	nein	ja	nein	negativ
17100196	60	m	nein	nein	nein	ja	negativ
17100197	29	m	nein	nein	ja	ja	negativ
17100198	51	m	nein	nein	ja	nein	negativ
17100199	34	w	nein	nein	nein	ja	negativ
17100200	31	m	ja	nein	ja	ja	negativ
17100201	43	m	nein	nein	ja	nein	negativ
17100202	38	m	ja	ja	ja	nein	negativ
17100203	32	m	nein	nein	ja	nein	negativ
17100204	49	m	ja	nein	ja	nein	negativ
17100205	40	m	ja	nein	ja	nein	negativ
17100206	54	m	nein	ja	ja	ja	negativ
17100207	31	m	ja	nein	ja	nein	negativ
17100208	29	m	nein	ja	nein	nein	negativ
17100209	42	m	nein	nein	ja	ja	negativ
17100210	24	m	ja	nein	ja	nein	negativ
17100211	46	m	ja	ja	ja	nein	negativ
17100212	34	m	ja	nein	ja	nein	negativ
17100213	31	m	ja	nein	ja	nein	negativ
17100214	27	m	nein	nein	ja	nein	negativ
17100215	42	m	ja	nein	ja	nein	negativ
17100216	27	w	ja	ja	ja	nein	negativ
17100217	27	m	nein	nein	ja	nein	negativ
17100218	45	m	nein	nein	nein	ja	negativ
17100219	26	m	nein	nein	ja	nein	negativ
17100220	21	m	ja	nein	nein	nein	negativ
17100221	24	m	ja	ja	ja	ja	negativ
17100223	25	m	ja	nein	nein	nein	negativ
17100224	30	m	ja	ja	ja	nein	negativ
17100225	59	m	nein	nein	ja	nein	negativ
17100226	54	m	nein	nein	nein	nein	negativ
17100227	54	m	nein	nein	ja	nein	negativ
17100228	26	m	nein	nein	ja	nein	negativ
17100229	46	m	nein	nein	nein	ja	negativ
17100230	35	m	ja	ja	ja	nein	negativ
17100231	36	m	ja	nein	ja	ja	negativ
17100232	40	m	ja	ja	ja	nein	negativ
17100233	34	w	nein	nein	nein	ja	negativ
17100234	33	m	nein	nein	ja	nein	negativ
17100235	24	m	nein	nein	ja	nein	negativ
17100236	26	m	nein	nein	ja	nein	negativ
17100237	52	m	nein	nein	nein	ja	negativ
17100238	51	m	nein	nein	ja	nein	negativ
17100239	50	w	nein	nein	nein	ja	negativ
17100240	40	m	nein	nein	nein	ja	negativ

17100241	62	w	nein	nein	nein	ja	negativ
17100242	29	m	nein	nein	ja	nein	negativ
17100243	35	m	ja	ja	ja	nein	negativ
17100244	49	m	ja	nein	ja	nein	negativ
17100245	26	m	ja	ja	ja	nein	negativ
17100246	31	m	ja	ja	ja	nein	negativ
17100247	23	m	ja	ja	ja	nein	negativ
17100248	30	m	nein	ja	ja	nein	negativ
17100249	28	m	ja	ja	ja	nein	negativ
17100250	42	m	ja	ja	ja	nein	negativ
17100251	26	m	ja	ja	ja	nein	negativ
17100252	40	m	ja	ja	ja	nein	negativ
17100253	24	m	ja	ja	ja	nein	negativ
17100254	53	m	nein	ja	nein	nein	negativ
17100256	30	m	ja	ja	ja	nein	negativ
17100257	34	m	ja	ja	ja	nein	negativ
17100258	25	m	nein	ja	nein	nein	negativ
17100259	50	m	ja	ja	ja	nein	negativ
17100260	47	m	ja	ja	ja	nein	negativ
17100261	47	m	ja	ja	ja	nein	negativ
17100262	26	m	ja	ja	ja	ja	negativ
17100263	28	m	ja	nein	ja	nein	negativ
17100264	24	m	ja	ja	ja	nein	negativ
17100265	30	m	ja	ja	ja	nein	negativ
17100266	29	m	ja	nein	ja	nein	negativ
17100267	30	m	ja	ja	ja	nein	negativ
17100268	29	m	ja	ja	ja	nein	negativ
17100269	27	m	ja	ja	ja	nein	negativ
17100270	32	m	ja	ja	ja	nein	negativ
17100271	44	m	ja	ja	ja	nein	negativ
17100272	26	m	ja	ja	ja	nein	negativ
17100273	31	m	ja	ja	ja	nein	negativ
17100274	33	m	ja	ja	ja	nein	negativ
17100275	29	m	ja	ja	ja	nein	negativ
17100276	43	m	ja	ja	ja	nein	negativ
17100277	39	m	ja	ja	ja	nein	negativ
17100278	36	w	nein	nein	nein	ja	negativ
17100279	27	m	ja	ja	ja	nein	negativ
17100280	28	m	ja	ja	ja	nein	negativ
17100281	52	m	nein	nein	nein	ja	negativ
17100282	26	m	ja	ja	ja	nein	negativ
17100283	43	m	ja	ja	ja	nein	negativ
17100284	45	m	ja	ja	ja	nein	negativ
17100285	37	m	ja	ja	ja	nein	negativ
17100286	52	m	ja	ja	ja	nein	negativ
17100287	42	m	ja	ja	ja	nein	negativ
17100288	30	m	ja	ja	ja	nein	negativ
17100289	37	m	ja	ja	ja	nein	negativ
17100290	40	m	ja	ja	ja	nein	negativ
17100291	58	m	nein	nein	nein	ja	negativ

17100292	43	m	nein	nein	nein	ja	negativ
17100293	61	m	nein	nein	nein	ja	negativ
17100294	51	m	nein	nein	nein	ja	negativ
17100295	30	w	nein	nein	nein	ja	negativ
17100296	22	w	nein	nein	nein	ja	negativ
17100297	44	m	nein	nein	nein	ja	negativ
17100298	30	m	ja	ja	ja	nein	negativ
17100299	28	m	ja	ja	ja	nein	negativ
17100300	26	m	nein	nein	nein	ja	negativ
17100301	46	m	nein	nein	ja	ja	negativ
17100302	28	m	nein	nein	nein	ja	negativ
17100303	24	w	nein	nein	nein	ja	negativ
17100304	27	m	ja	nein	ja	nein	negativ
17100305	40	m	ja	ja	ja	nein	negativ
17100306	52	m	nein	nein	nein	ja	negativ
17100307	21	w	ja	nein	nein	nein	negativ
17100308	36	m	ja	nein	ja	nein	negativ
17100309	22	m	ja	nein	nein	nein	negativ
17100310	23	m	ja	nein	nein	nein	negativ
17100311	34	m	ja	nein	nein	nein	negativ
17100312	21	m	ja	nein	nein	nein	negativ
17100313	32	m	ja	nein	nein	nein	negativ
17100314	29	m	ja	nein	ja	nein	negativ
17100315	29	m	ja	nein	nein	nein	negativ
17100316	18	w	ja	nein	nein	nein	negativ
17100317	21	w	ja	nein	nein	nein	negativ
17100318	47	m	ja	nein	ja	nein	negativ
17100319	49	w	ja	nein	nein	nein	negativ
17100320	35	m	ja	nein	nein	nein	negativ
17100321	53	m	ja	nein	ja	nein	negativ
17100322	19	m	ja	nein	nein	nein	negativ
17100323	35	m	ja	nein	ja	nein	negativ
17100324	38	m	ja	nein	ja	nein	negativ
17100325	21	m	ja	nein	nein	nein	negativ
17100326	48	m	ja	nein	ja	nein	negativ
17100327	48	m	ja	nein	ja	nein	negativ
17100328	28	m	ja	nein	ja	nein	negativ
17100329	50	m	ja	nein	ja	nein	negativ
17100330	26	w	ja	nein	nein	nein	negativ
17100331	53	m	ja	nein	nein	nein	negativ
17100332	25	m	ja	nein	ja	nein	negativ
17100333	18	m	ja	nein	nein	nein	negativ
17100334	29	m	ja	nein	ja	nein	negativ
17100335	21	m	ja	nein	nein	nein	negativ
17100336	19	w	ja	nein	nein	nein	negativ
17100337	20	w	ja	nein	nein	nein	negativ
17100338	43	m	ja	nein	ja	nein	negativ
17100339	24	m	ja	nein	nein	nein	negativ
17100340	37	m	ja	nein	nein	ja	negativ
17100341	25	m	ja	nein	nein	nein	negativ

17100342	52	m	ja	nein	nein	nein	negativ
17100343	44	m	ja	nein	ja	nein	negativ
17100344	50	m	ja	nein	nein	nein	negativ
17100345	41	m	ja	nein	ja	nein	negativ
17100346	35	m	ja	nein	ja	nein	negativ
17100347	25	w	ja	nein	nein	nein	negativ
17100348	21	w	ja	nein	nein	nein	negativ
17100349	27	w	nein	nein	nein	ja	negativ
17100350	23	w	nein	nein	nein	ja	negativ
17100351	27	m	ja	nein	ja	nein	negativ
17100352	52	w	ja	nein	nein	nein	negativ
17100353	45	m	nein	nein	nein	ja	negativ
17100354	37	w	nein	nein	nein	ja	negativ
17100355	25	m	ja	nein	nein	nein	negativ
17100356	29	m	ja	nein	nein	nein	negativ
17100357	21	m	ja	nein	nein	nein	negativ
17100358	21	w	ja	nein	nein	nein	negativ
17100359	23	m	nein	nein	ja	nein	negativ
17100360	23	w	ja	nein	nein	nein	negativ
17100361	28	m	ja	nein	ja	nein	negativ
17100362	25	m	ja	nein	nein	nein	negativ
17100363	34	m	ja	nein	nein	nein	negativ
17100364	34	m	ja	nein	ja	nein	negativ
17100365	45	m	ja	ja	ja	nein	negativ
17100366	30	m	ja	nein	nein	nein	negativ
17100367	53	m	nein	nein	ja	nein	negativ
17100368	38	w	ja	nein	nein	nein	negativ
17100369	30	m	ja	nein	ja	nein	negativ
17100371	43	m	ja	nein	ja	nein	negativ
17100372	21	m	ja	nein	nein	nein	negativ
17100373	28	m	ja	nein	ja	nein	negativ
17100374	23	m	ja	nein	nein	nein	negativ
17100375	26	m	ja	nein	nein	nein	negativ
17100376	39	m	ja	nein	ja	nein	negativ
17100377	19	w	ja	nein	nein	nein	negativ
17100378	23	m	ja	nein	nein	nein	negativ
17100379	53	m	ja	nein	nein	nein	negativ
17100380	28	m	ja	nein	nein	nein	negativ
17100381	23	m	ja	nein	nein	nein	negativ
17100382	21	m	ja	nein	nein	nein	negativ
17100383	23	w	ja	nein	nein	nein	negativ
17100384	21	m	ja	nein	nein	nein	negativ
17100385	29	m	ja	nein	ja	nein	negativ
17100386	27	m	ja	nein	nein	nein	negativ
17100388	38	m	ja	nein	nein	nein	negativ
17100389	42	m	ja	nein	ja	nein	negativ
17100390	46	m	ja	nein	ja	nein	negativ
17100391	21	m	ja	nein	nein	nein	negativ
17100392	24	m	ja	nein	nein	nein	negativ
17100393	58	m	ja	nein	ja	nein	negativ

17100394	28	m	ja	nein	ja	nein	negativ
17100396	38	m	ja	nein	ja	nein	negativ
17100397	29	m	ja	ja	ja	nein	negativ
17100398	45	m	ja	nein	ja	nein	negativ
17100399	25	m	ja	nein	nein	nein	negativ
17100400	33	m	ja	nein	ja	nein	negativ
17100401	35	m	ja	nein	nein	nein	negativ
17100402	56	m	ja	nein	ja	nein	negativ
17100403	39	m	ja	nein	ja	nein	negativ
17100404	48	m	ja	nein	ja	nein	negativ
17100405	36	m	ja	nein	ja	nein	negativ
17100406	19	m	ja	nein	nein	nein	negativ
17100407	22	w	nein	nein	nein	ja	negativ
17100408	24	m	ja	nein	nein	nein	negativ
17100409	29	m	ja	nein	ja	nein	negativ
17100410	23	m	ja	nein	nein	nein	negativ
17100411	44	m	ja	nein	ja	nein	negativ
17100412	18	m	ja	nein	nein	nein	negativ
17100413	35	m	ja	nein	nein	nein	negativ
17100414	26	m	ja	nein	nein	nein	negativ
17100415	21	m	ja	nein	nein	nein	negativ
17100416	37	m	ja	nein	nein	nein	negativ
17100417	22	w	ja	nein	nein	nein	negativ
17100418	26	m	nein	nein	nein	ja	negativ
17100419	31	m	ja	nein	nein	nein	negativ
17100421	49	m	ja	nein	ja	nein	negativ
17100422	32	m	ja	nein	ja	nein	negativ
17100423	46	m	ja	ja	ja	nein	negativ
17100424	33	m	ja	nein	ja	nein	negativ
17100425	31	m	ja	nein	ja	nein	negativ
17100426	27	m	ja	nein	nein	nein	negativ
17100427	26	m	ja	nein	nein	nein	negativ
17100428	26	m	ja	nein	nein	nein	negativ
17100429	54	m	ja	nein	nein	nein	negativ
17100430	40	m	ja	nein	ja	nein	negativ
17100431	35	m	ja	ja	ja	nein	negativ
17100432	43	m	ja	nein	ja	nein	negativ
17100433	21	m	ja	nein	nein	nein	negativ
17100434	21	w	ja	nein	nein	nein	negativ
17100435	43	m	ja	nein	nein	nein	negativ
17100436	54	m	ja	nein	ja	nein	negativ
17100437	45	m	ja	nein	nein	ja	negativ
17100438	26	m	ja	nein	nein	nein	negativ
17100439	28	m	ja	nein	ja	nein	negativ
17100440	25	m	ja	ja	ja	nein	negativ
17100441	28	m	ja	nein	ja	nein	negativ
17100442	33	m	ja	nein	ja	nein	negativ
17100443	38	m	nein	nein	nein	ja	negativ
17100444	22	w	nein	nein	nein	ja	negativ
17100445	57	m	nein	nein	nein	ja	negativ

17100446	50	w	nein	nein	nein	ja	negativ
17100447	25	m	nein	nein	nein	ja	negativ
17100448	50	w	nein	nein	nein	ja	negativ
17100449	54	w	nein	nein	nein	ja	negativ
17100450	24	w	nein	nein	nein	ja	negativ
17100451	49	m	nein	nein	nein	ja	negativ
17100452	36	m	nein	nein	ja	ja	negativ
17100453	53	m	nein	nein	nein	ja	negativ
17100454	50	m	nein	nein	nein	ja	negativ
17100455	60	w	nein	nein	nein	ja	negativ
17100456	57	m	nein	nein	nein	ja	negativ
17100457	52	w	nein	nein	nein	ja	negativ
17100459	53	m	nein	nein	nein	ja	negativ
17100460	37	w	nein	nein	nein	ja	negativ
17100461	45	m	nein	nein	nein	ja	negativ
17100462	25	w	nein	nein	nein	ja	negativ
17100463	49	m	nein	nein	nein	ja	negativ
17100464	38	w	nein	nein	nein	ja	negativ
17100465	22	w	nein	nein	nein	ja	negativ
17100466	23	w	nein	nein	nein	ja	negativ
17100467	46	m	nein	nein	nein	ja	negativ
17100468	52	m	nein	nein	nein	ja	negativ
17100469	54	m	nein	nein	nein	ja	negativ
17100470	41	m	nein	nein	nein	ja	negativ
17100471	47	w	nein	nein	nein	ja	negativ
17100472	30	w	nein	nein	nein	ja	negativ
17100473	57	m	nein	nein	nein	ja	negativ
17100474	26	w	nein	nein	nein	ja	negativ
17100475	24	m	ja	nein	nein	ja	negativ
17100476	30	m	nein	nein	ja	ja	negativ
17100477	42	w	nein	nein	nein	ja	negativ
17100478	54	m	nein	nein	nein	ja	negativ
17100479	43	m	nein	nein	nein	ja	negativ
17100480	60	w	nein	nein	nein	ja	negativ
17100481	30	m	nein	nein	nein	ja	negativ
17100482	59	m	nein	nein	nein	ja	negativ
17100483	44	m	nein	nein	nein	ja	negativ
17100484	22	w	nein	nein	nein	ja	negativ
17100485	57	m	nein	nein	nein	ja	negativ
17100486	63	m	nein	nein	nein	ja	negativ
17100487	53	w	nein	nein	nein	ja	negativ
17100488	50	m	nein	nein	nein	ja	negativ
17100489	53	m	nein	nein	nein	ja	negativ
17100490	23	w	ja	nein	nein	nein	negativ
17100491	58	m	ja	nein	ja	nein	negativ
17100492	22	w	ja	nein	nein	nein	negativ
17100493	19	m	ja	nein	nein	nein	negativ
17100494	20	m	ja	nein	nein	nein	negativ
17100495	38	m	ja	nein	ja	nein	negativ
17100496	28	m	ja	nein	nein	nein	negativ

17100497	54	m	ja	nein	nein	nein	negativ
17100498	55	m	ja	nein	nein	nein	negativ
17100499	37	m	ja	nein	ja	ja	negativ
17100500	24	m	ja	nein	nein	nein	negativ
17100501	25	m	ja	nein	nein	nein	negativ
17100502	27	w	ja	nein	nein	nein	negativ
17100503	53	m	ja	nein	ja	nein	negativ
17100504	19	m	ja	nein	nein	nein	negativ
17100505	19	w	ja	nein	nein	nein	negativ
17100506	32	m	ja	nein	ja	nein	negativ
17100507	41	m	ja	nein	nein	nein	negativ
17100508	46	m	ja	nein	nein	nein	negativ
17100509	20	m	ja	nein	nein	nein	negativ
17100510	34	m	ja	nein	ja	nein	negativ
17100511	19	m	ja	nein	nein	nein	negativ
17100512	22	m	ja	nein	nein	nein	negativ
17100513	37	w	ja	nein	nein	nein	negativ
17100514	49	m	ja	ja	ja	nein	negativ
17100515	20	m	ja	nein	nein	nein	negativ
17100516	25	m	ja	nein	nein	nein	negativ
17100517	47	m	ja	nein	ja	nein	negativ
17100518	31	m	ja	nein	ja	nein	negativ
17100519	45	w	ja	nein	nein	nein	negativ
17100520	20	w	ja	nein	nein	nein	negativ
17100521	51	m	ja	nein	ja	nein	negativ
17100522	32	m	ja	nein	nein	nein	negativ
17100523	19	w	ja	nein	nein	nein	negativ
17100524	32	m	ja	nein	ja	nein	negativ
17100525	36	w	ja	nein	nein	nein	negativ
17100526	35	m	ja	nein	nein	nein	negativ
17100527	57	m	ja	nein	nein	nein	negativ
17100528	45	m	ja	nein	ja	nein	negativ
17100529	36	m	ja	ja	ja	nein	negativ
17100530	41	m	ja	nein	nein	nein	negativ
17100531	38	m	ja	nein	ja	nein	negativ
17100532	30	m	ja	nein	ja	nein	negativ
17100533	29	m	ja	nein	nein	nein	negativ
17100534	29	m	ja	nein	ja	nein	negativ
17100535	18	m	ja	nein	nein	nein	negativ
17100536	22	w	ja	nein	nein	nein	negativ
17100537	25	m	ja	nein	ja	nein	negativ
17100538	24	m	ja	nein	nein	nein	negativ
17100539	25	m	ja	nein	nein	nein	negativ
17100540	33	m	ja	ja	ja	nein	negativ
17100541	49	m	ja	nein	ja	nein	negativ
17100542	43	m	ja	nein	ja	nein	negativ
17100543	33	w	nein	nein	nein	ja	negativ
17100544	26	w	nein	nein	nein	ja	negativ
17100545	37	w	nein	nein	nein	ja	negativ
17100546	53	m	nein	nein	nein	ja	negativ

17100547	25	w	nein	nein	nein	ja	negativ
17100548	39	m	nein	nein	nein	ja	negativ
17100549	35	w	nein	nein	nein	ja	negativ
17100550	46	m	nein	nein	nein	ja	negativ
17100551	75	m	nein	nein	nein	ja	negativ
17100552	38	w	nein	nein	nein	ja	negativ
17100553	42	w	nein	nein	nein	ja	negativ
17100554	37	w	nein	nein	nein	ja	negativ
17100555	55	w	nein	nein	nein	ja	negativ
17100556	44	m	ja	nein	nein	nein	negativ
17100557	41	m	nein	nein	nein	ja	negativ
17100558	54	m	nein	nein	nein	ja	negativ
17100559	45	m	nein	nein	nein	ja	negativ
17100560	22	m	nein	nein	nein	ja	negativ
17100562	53	w	nein	nein	nein	ja	negativ
17100563	48	m	nein	nein	nein	ja	negativ
17100564	42	w	nein	nein	nein	ja	negativ
17100565	64	m	nein	nein	nein	ja	negativ
17100567	57	m	nein	nein	nein	ja	negativ
17100568	55	w	nein	nein	nein	ja	negativ
17100569	34	m	ja	nein	nein	ja	negativ
17100570	28	m	nein	nein	nein	ja	negativ
17100571	47	w	nein	nein	nein	ja	negativ
17100572	28	w	Nein	Nein	Nein	Ja	negativ
17100573	59	w	nein	nein	nein	ja	negativ
17100574	62	w	nein	nein	nein	nein	negativ
17100575	23	w	nein	nein	nein	ja	negativ
17100576	30	w	nein	nein	nein	ja	negativ
17100577	54	w	nein	nein	nein	ja	negativ
17100578	40	m	nein	nein	nein	ja	negativ
17100579	24	w	nein	nein	nein	ja	negativ
17100580	21	m	nein	nein	nein	ja	negativ
17100581	37	w	nein	nein	nein	nein	negativ
17100582	59	w	nein	nein	nein	nein	negativ
17100583	50	m	ja	nein	ja	nein	negativ
17100584	23	w	ja	nein	nein	nein	negativ
17100585	26	m	ja	nein	ja	nein	negativ
17100586	58	m	ja	nein	ja	nein	negativ
17100587	26	m	ja	nein	nein	nein	negativ
17100588	31	m	ja	nein	ja	ja	negativ
17100589	50	w	nein	nein	nein	ja	negativ
17100590	40	m	ja	ja	ja	nein	negativ
17100591	26	m	ja	nein	ja	nein	negativ
17100592	47	m	ja	nein	ja	nein	negativ
17100593	54	m	nein	nein	nein	ja	negativ
17100594	39	m	ja	nein	ja	nein	negativ
17100595	27	m	ja	nein	ja	nein	negativ
17100596	30	w	nein	nein	nein	ja	negativ
17100597	28	m	ja	nein	ja	nein	negativ
17100598	24	m	ja	nein	nein	nein	negativ

17100599	43	m	ja	nein	ja	nein	negativ
17100600	53	m	ja	nein	ja	nein	negativ
17100601	26	m	nein	nein	ja	nein	negativ
17100602	33	m	ja	nein	nein	nein	negativ
17100603	45	m	ja	nein	ja	nein	negativ
17100604	33	m	ja	nein	ja	nein	negativ
17100605	25	m	ja	ja	ja	nein	negativ
17100606	44	m	ja	nein	nein	nein	negativ
17100607	32	w	ja	ja	nein	nein	negativ
17100608	52	m	ja	nein	nein	nein	negativ
17100609	44	m	ja	nein	nein	nein	negativ
17100610	42	m	ja	nein	ja	nein	negativ
17100611	47	m	ja	nein	nein	nein	negativ
17100612	46	m	ja	ja	ja	nein	negativ
17100613	45	m	ja	ja	ja	ja	negativ
17100614	56	m	nein	nein	nein	ja	negativ
17100615	31	m	ja	ja	ja	nein	negativ
17100616	35	m	nein	nein	ja	ja	negativ
17100617	51	m	nein	nein	nein	ja	negativ
17100618	31	m	ja	nein	ja	nein	negativ
17100619	57	m	nein	nein	ja	ja	negativ
17100620	54	m	ja	nein	ja	nein	negativ
17100621	31	m	ja	nein	ja	nein	negativ
17100622	25	m	ja	nein	nein	nein	negativ
17100623	52	w	nein	nein	nein	ja	negativ
17100624	42	m	ja	nein	ja	nein	negativ
17100625	48	m	ja	nein	ja	nein	negativ
17100626	50	m	ja	nein	ja	nein	negativ
17100627	57	m	nein	nein	nein	ja	negativ
17100628	38	m	ja	nein	ja	nein	negativ
17100629	56	m	nein	nein	nein	ja	negativ
17100630	27	m	ja	ja	ja	ja	negativ
17100631	28	m	ja	nein	nein	nein	negativ
17100632	28	m	ja	nein	ja	nein	negativ
17100633	25	m	nein	nein	ja	nein	negativ
17100635	41	m	ja	nein	ja	nein	negativ
17100636	27	m	ja	nein	ja	nein	negativ
17100637	20	m	ja	nein	nein	nein	negativ
17100638	25	m	ja	nein	ja	nein	negativ
17100639	27	m	ja	nein	ja	nein	negativ
17100640	25	m	ja	ja	ja	nein	negativ
17100641	32	m	ja	nein	ja	nein	negativ
17100642	30	m	ja	nein	ja	nein	negativ
17100643	23	m	nein	nein	nein	ja	negativ
17100644	42	m	ja	nein	ja	nein	negativ
17100645	43	m	ja	ja	ja	nein	negativ
17100646	46	m	ja	ja	ja	nein	negativ
17100647	33	m	ja	nein	ja	ja	negativ
17100648	29	m	ja	ja	ja	ja	negativ
17100649	55	m	nein	nein	ja	nein	negativ

17100650	43	w	ja	nein	nein	nein	negativ
17100651	53	m	nein	nein	nein	ja	negativ
17100652	34	m	ja	nein	ja	nein	negativ
17100653	49	m	ja	nein	nein	nein	negativ
17100654	45	m	ja	nein	ja	nein	negativ
17100655	21	m	ja	nein	nein	nein	negativ
17100656	23	m	ja	nein	nein	nein	negativ
17100657	24	m	ja	nein	nein	nein	negativ
17100658	30	w	nein	nein	nein	ja	negativ
17100659	29	m	ja	ja	ja	nein	negativ
17100660	27	m	ja	ja	ja	nein	negativ
17100661	41	m	ja	nein	ja	nein	negativ
17100662	35	m	ja	ja	ja	nein	negativ
17100663	41	m	ja	ja	ja	ja	negativ
17100664	25	m	ja	ja	ja	nein	negativ
17100665	25	m	ja	ja	ja	nein	negativ
17100666	21	m	ja	nein	ja	nein	negativ
17100667	33	m	ja	ja	ja	nein	negativ
17100668	31	m	ja	nein	ja	nein	negativ
17100669	34	m	ja	ja	ja	nein	negativ
17100670	26	m	nein	nein	nein	ja	negativ
17100671	31	m	ja	ja	ja	nein	negativ
17100672	33	m	ja	nein	nein	nein	negativ
17100673	45	m	ja	ja	ja	nein	negativ
17100674	52	m	nein	nein	ja	nein	negativ
17100675	23	m	nein	nein	nein	ja	negativ
17100676	32	m	ja	ja	ja	nein	negativ
17100677	35	m	nein	nein	ja	ja	negativ
17100678	31	m	ja	ja	ja	nein	negativ
17100679	39	m	ja	nein	ja	nein	negativ
17100680	26	m	ja	ja	ja	nein	negativ
17100681	25	m	ja	nein	ja	nein	negativ
17100682	50	m	nein	nein	ja	nein	negativ
17100683	51	m	ja	nein	ja	nein	negativ
17100684	57	m	nein	nein	ja	nein	negativ
17100685	37	m	nein	nein	ja	nein	negativ
17100686	46	m	nein	nein	ja	nein	negativ
17100687	36	m	ja	ja	ja	nein	negativ
17100688	54	m	nein	nein	ja	nein	negativ
17100689	55	m	ja	nein	ja	ja	negativ
17100690	50	m	ja	nein	ja	ja	negativ
17100691	43	m	nein	nein	ja	ja	negativ
17100692	43	m	nein	nein	ja	nein	negativ
17100693	32	w	nein	nein	ja	ja	negativ
17100694	52	m	nein	nein	ja	ja	negativ
17100695	45	m	ja	nein	nein	nein	negativ
17100696	57	m	nein	nein	ja	nein	negativ
17100697	33	m	nein	nein	ja	nein	negativ
17100698	31	m	nein	nein	nein	ja	negativ
17100699	56	w	nein	nein	nein	ja	negativ

17100700	43	w	nein	nein	nein	ja	negativ
17100701	22	m	ja	nein	ja	ja	negativ
17100702	46	m	nein	nein	nein	ja	negativ
17100703	55	m	nein	nein	nein	ja	negativ
17100704	57	m	nein	nein	nein	ja	negativ
17100705	27	m	ja	ja	ja	nein	negativ
17100706	43	m	nein	nein	ja	ja	negativ
17100707	40	m	ja	nein	ja	nein	negativ
17100708	44	m	nein	nein	ja	nein	negativ
17100709	43	m	ja	nein	ja	nein	negativ
17100710	30	m	nein	nein	ja	ja	negativ
17100711	38	m	nein	nein	ja	nein	negativ
17100712	53	m	nein	nein	ja	nein	negativ
17100713	38	m	nein	nein	ja	ja	negativ
17100714	49	m	nein	nein	ja	nein	negativ
17100715	56	m	nein	nein	ja	nein	negativ
17100716	23	m	nein	nein	ja	ja	negativ
17100717	54	m	nein	nein	ja	nein	negativ
17100718	42	m	nein	nein	ja	nein	negativ
17100719	40	m	nein	nein	ja	nein	negativ
17100720	39	m	nein	nein	ja	nein	negativ
17100721	50	m	ja	nein	ja	nein	negativ
17100722	44	m	nein	nein	ja	ja	negativ
17100723	50	m	nein	nein	ja	nein	negativ
17100724	41	m	ja	ja	ja	nein	negativ
17100725	53	m	ja	nein	ja	nein	negativ
17100726	51	m	ja	nein	ja	nein	negativ
17100727	23	m	ja	nein	ja	nein	negativ
17100728	25	m	ja	ja	ja	nein	negativ
17100729	32	w	nein	nein	nein	ja	negativ
17100730	39	m	ja	nein	ja	nein	negativ
17100731	25	m	ja	ja	ja	nein	negativ
17100732	56	m	ja	ja	ja	nein	negativ
17100734	32	m	ja	nein	nein	nein	negativ
17100735	23	m	ja	ja	ja	nein	negativ
17100736	28	m	ja	ja	ja	nein	negativ
17100737	53	m	nein	nein	nein	ja	negativ
17100738	25	m	ja	nein	ja	nein	negativ
17100739	26	m	nein	nein	nein	ja	negativ
17100740	27	m	ja	nein	ja	nein	negativ
17100741	21	m	ja	nein	nein	nein	negativ
17100742	28	w	ja	nein	nein	nein	negativ
17100743	29	w	ja	nein	nein	nein	negativ
